# Extracellular BCL2 Proteins Are Danger-Associated Molecular Patterns That Reduce Tissue Damage in Murine Models of Ischemia-Reperfusion Injury

**DOI:** 10.1371/journal.pone.0009103

**Published:** 2010-02-08

**Authors:** Akiko Iwata, Vicki Morgan-Stevenson, Barbara Schwartz, Li Liu, Joan Tupper, Xiaodong Zhu, John Harlan, Robert Winn

**Affiliations:** 1 Department of Surgery, University of Washington, Seattle, Washington, United States of America; 2 Department of Medicine, University of Washington, Seattle, Washington, United States of America; Charité-Universitätsmedizin Berlin, Germany

## Abstract

**Background:**

Ischemia-reperfusion (I/R) injury contributes to organ dysfunction in a variety of clinical disorders, including myocardial infarction, stroke, organ transplantation, and hemorrhagic shock. Recent investigations have demonstrated that apoptosis as an important mechanism of cell death leading to organ dysfunction following I/R. Intracellular danger-associated molecular patterns (DAMPs) released during cell death can activate cytoprotective responses by engaging receptors of the innate immune system.

**Methodology/Principal Findings:**

Ischemia was induced in the mouse hind limb by tourniquet or in the heart by coronary artery ligation. Reperfusion injury of skeletal or cardiac muscle was markedly reduced by intraperitoneal or subcutaneous injection of recombinant human (rh)BCL2 protein or rhBCL2-related protein A1 (BCL2A1) (50 ng/g) given prior to ischemia or at the time of reperfusion. The cytoprotective activity of extracellular rhBCL2 or rhBCL2A1 protein was mapped to the BH4 domain, as treatment with a mutant BCL2 protein lacking the BH4 domain was not protective, whereas peptides derived from the BH4 domain of BCL2 or the BH4-like domain of BCL2A1 were. Protection by extracellular rhBCL2 or rhBCL2A1 was associated with a reduction in apoptosis in skeletal and cardiac muscle following I/R, concomitant with increased expression of endogenous mouse BCL2 (mBCL2) protein. Notably, treatment with rhBCL2A1 protein did not protect mice deficient in toll-like receptor-2 (TLR2) or the adaptor protein, myeloid differentiation factor-88 (MyD88).

**Conclusions/Significance:**

Treatment with cytokine-like doses of rhBCL2 or rhBCL2A1 protein or BH4-domain peptides reduces apoptosis and tissue injury following I/R by a TLR2-MyD88-dependent mechanism. These findings establish a novel extracellular cytoprotective activity of BCL2 BH4-domain proteins as potent cytoprotective DAMPs.

## Introduction

Cellular injury resulting from tissue damage induced by a variety of insults elicits host defense and repair responses. With primary necrosis or secondary necrosis (when apoptotic cells are not cleared by phagocytosis) intracellular constituents, nuclear as well as cytoplasmic, are released extracellularly. In the extracellular environment these intracellular constituents may act as danger-associated molecular patterns (DAMPs) or ‘alarmins’, engaging receptors on immune or adjacent cells and triggering activation of the innate immune system (reviewed in [1]–[3]). Under some circumstances, pro-inflammatory responses to intracellular DAMPs may exacerbate tissue injury, whereas under other conditions DAMPs may elicit cytoprotective responses.

The BCL2 family consists of anti- and pro-apoptotic proteins that have been extensively studied as intracellular regulators of cell death (reviewed in [4]). In previous studies in a murine model of sepsis induced by cecal ligation and puncture (CLP), we found that intraperitoneal (ip) instillation of myeloid cells over-expressing BCL2 protected normal mice against death following CLP [5]. Since the adoptively transferred cells eventually undergo necrosis or apoptosis with secondary necrosis, we examined the possibility that BCL2 protein itself was the transferred protective factor. Indeed, we found that administration of BCL2 protein alone was sufficient to reduce sepsis-induced lethality (manuscript in preparation). The experiments described herein demonstrate a novel extracellular cytoprotective function against severe I/R injury for two anti-apoptotic members of the BCL2 family, BCL2 and BCL2-related protein A1 (BCL2A1), which have previously been thought to function only intracellularly. We show that exogenously administered recombinant human BCL2 (rhBCL2) or rhBCL2A1 protein and synthetic peptides derived from the BH4 domain of BCL2 or the BH4-like domain of BCL2A1 [6] confer protection against I/R injury of hind limb skeletal muscle or cardiac muscle. This protection was associated with increased expression of endogenous mouse BCL2 protein and reduced apoptosis in the muscle subjected to I/R injury. The protection afforded by rhBCL2A1 protein was not seen in mice deficient in either toll-like receptor-2 (TLR2) or the adaptor molecule MyD88, indicating that the protective pathway required TLR2-MyD88 signaling. We propose that BCL2, BCL2A1, and BH4-domain peptides are cytoprotective DAMPs.

## Materials and Methods

### Animals

All protocols were approved by the Animal Care and Use Committee of the University of Washington and complied with the NIH guidelines for care and use of animals. Mice were maintained at the University of Washington, Department of Comparative Medicine facility. Sprague-Dawley rats and C57BL/6 mice were obtained from Charles River Breeding Laboratory (Wilmington, MA). C3H/HeJ mice were obtained from Jackson Laboratory (Bar Harbor, ME). TLR2^−/−^ and MyD88^−/−^ mice were bred in the animal facility at the University of Washington and breeding pairs were a gift of Dr. S. Akira (Osaka, Japan).

### Proteins and Peptides

rhBCL2, rhUbiquitin, rhBIM (BCL2-like 11; BCL2L11), and rhBCL2A1 were purchased from R&D Systems (Minneapolis, MN). Mutant rhBCL2 (-BH4) and rhBAX (BCL2-associated X) were purchased from ProteinX (San Diego, CA). rhBCL2 protein consisted of amino acids 1–212 (accession number P10415) of human Bcl-2, thus the carboxyl terminal was deleted and replaced with a ten histidine tag. rhBCL2A1 (BCL2-related protein A1) protein consisted of amino acids 1–152 of human BCL2-related protein BCL2A1 (accession number NP004040) missing the carboxyl terminal hydrophobic domain that was replaced with a six histidine tag. rhUbiquitn consisted of amino acids 1–76 (accession number CAA28495.1) containing the amino acids ATVID followed by a 10 histidine tag at its amino terminal. rhBim consisted of amino acids 2–120 (accession number AAC39594) of human Bim long, with a deleted carboxyl terminal transmembrane domain replaced with a six histidine tag. Purity of the proteins was >95% for the recombinant proteins as determined by SDS-PAGE and visualized by silver stain per manufacturer (R&D Systems). Endotoxin concentration in the recombinant protein was less than 0.15 EU/mg protein (R&D Systems).

BCL2-BH4 peptide consisted of amino acid 7–31 (TGYDNREIVMKYIHYKLSQRGYEWD-CONH2 (SynPep, Dublin, CA)). BCL2A1-BH4 peptide consisted of amino acids 6–30 (FGYIYRLAQDYLQCVLQIPQPGSGP-CONH2 (SynPep)). BAK-BH3 peptide consisted of amino acids 72–87 (GQVGRQLAIIGDDIN; R&D Systems). Scrambled peptide had the sequence TWHMYGNQRDYIGDRSKIVYKLEYE-CONH2 (SynPep).

### Hind Limb I/R

In a previous study, we investigated anti-adhesion therapy in I/R injury of mouse hind limb skeletal muscle. We found that anti-adhesion therapy afforded significant protection against brief ischemia (30–60 minutes) followed by reperfusion [7]. Surprisingly, only a modest extension of ischemic time to 90 minutes produced a marked increase in muscle injury following reperfusion. Importantly, anti-adhesion therapy failed to protect against I/R injury with 90 minutes of ischemia, whereas anti-apoptotic therapy with a broad-spectrum caspase inhibitor was protective. These results indicated that tissue injury with prolonged ischemia was due to apoptosis rather than to leukocyte-mediated inflammation [7]. We proposed that brief ischemia was not an accurate model of human disease, and therefore we have used only prolonged ischemia in the current studies of hind limb I/R.

I/R of hind limbs was accomplished by cross-clamping of the aorta [7] in mice or by tourniquet [8] in mice and rats. Briefly, in the aorta cross-clamp experiments mice were pretreated with buprenorphine, anesthetized with halothane or isoflurane, a midline abdominal incision made, and the vena cava and aorta carefully separated. A small micro-vascular clamp was applied to the aorta distal to the renal arteries. The peritoneum and skin were then closed. For hind limb I/R induced by tourniquets in mice, bilateral tourniquets (Latex O-rings) were applied above the greater trochanter using the McGivney Hemorrhoid Ligator (Miltex, York, PA). Mice were maintained in a supine position under halothane or isoflurane in an incubator at 30°C during the 90-minute ischemic period. For hind limb I/R in rats, a #31 Sparco rubber band was placed as high on the thigh as possible on both legs and turned 7 times. The rats were maintained on isoflurane during the 90-minute period of ischemia.

The aortic clamp or the hind limb tourniquet was removed at the end of ischemia to establish reperfusion. With aorta clamping, the peritoneum and skin were closed, and mice were allowed to recover in the 30°C incubator in order to maintain normal body temperature. For both aorta clamping and tourniquet I/R, reperfusion was continued for 3 hours. The animals were then killed by exsanguination under ketamine anesthesia, and blood samples were taken for plasma creatine kinase (CK) determination by an automated analyzer. Muscle samples were taken from the left leg, fixed in 4% formalin, and processed for histological evaluation, including terminal deoxynucleotidyl transferase–mediated dUTP nick end-labeling (TUNEL) assay and immunocytochemistry (ICC).

In some experiments, reperfusion was continued for 24 hours and injury was assessed by mitochondrial function rather than CK release. Anesthetized mice were subjected to 90 minutes of tourniquet ischemia as described above followed by overnight reperfusion. Mice were treated by a subcutaneous injection of 1 µg of either rhBCL2A1 or rhBim at the time of reperfusion or after 4 hours of reperfusion. The following day, mice were killed, the previously ischemic muscle removed, and tissue viability measured using 3-(4,5-dimethylthiazol-2-yl)-2,5-diphenyl tetrazolium (MTT) [9]. Briefly, the excised muscle tissue was cut into pieces to increase the surface area and uptake of MTT. The tissue sections were placed in 3 ml of PBS supplemented with 60 ml of 5 mg/ml MTT and incubated for 3 hours at 37°C on a shaker. Samples were removed, blotted dry and the formazan salt extracted in 3 ml of 2-propanol overnight at 37°C in the dark. Absorbance at 570 nm of 200 µl of the solution was determined and normalized to the dry weight of the tissue. Viable mitochondria reduce MTT to a water-insoluble salt that is soluble in isopropanol and can be extracted by isopropanol. Live tissue with functional mitochondria will therefore have an increased optical density relative to dead tissue.

### Cardiac I/R

Two protocols were used for myocardial I/R. In protocol A, mice were treated with either rhBCL2 (1 µg/mouse) or rhBim (1 µg/mouse) protein given ip the day prior to myocardial I/R. The ischemic period was 60 minutes, followed by 2 hours of reperfusion. In protocol B, mice were treated with rhBCL2A1 (1 µg/mouse) or with rhBim (1 µg/mouse) given at the time of reperfusion, followed by 24 hours of reperfusion.

On the day of experiment, mice were pretreated with buprenorphine, anesthetized with isoflurane, their tracheae intubated, and placed on mechanical ventilation. A left thoracotomy was performed, the left anterior descending (LAD) artery located, and a ligature placed approximately 2–3 mm from the tip of the left auricle. A small piece of polyethylene tubing (PE-10) was used to secure the ligature taking care to prevent damage to the artery. Coronary occlusion and reperfusion were confirmed by visual inspection under a dissecting microscope by observing color changes of the tissue. Following the period of reperfusion specified by the protocol, mice were killed by exsanguination under anesthesia. The LAD was again ligated at original location and the aorta occluded. The infarct size was evaluated by double staining using 1.5% Evans blue dye (Sigma) and 1% triphenyltetrazolium chloride (TTC, Sigma). The area at risk was defined as the ratio of the area of the ischemic region to the left ventricular area, and the infarct size was defined as the ratio of the area of the infarct region to that of the ischemic region.

In separate experiments, mice pretreated with either rhBCL2 or rhBim were subjected to myocardial I/R. At the end of the reperfusion period, the heart was removed, fixed in formalin, and tissue sections cut and stained for TUNEL or mBCL2 protein.

### TUNEL and Immunocytochemistry (ICC)

Paraffin-embedded sections from leg muscle were examined for DNA strand breaks using the TUNEL technique (In Situ Cell Death Kit, Roche Molecular Biochemicals) as described by the manufacturer. Labeled endothelial, skeletal muscle, or smooth muscle cells were examined.

Skeletal or cardiac muscle was fixed in 4% formalin, paraffin embedded, and tissue sections cut. Tissue sections (5 µm) were subjected to antigen retrieval by boiling in citrate buffer (pH 6.0) in a microwave oven. Tissue was stained with a rabbit anti-mouse specific BCL2 polyclonal antibody (Upstate, Waltham, MA) or with active caspase-3 polyclonal antibody (Cell Signaling Technology, Danvers, MA). Immunoreactivity was detected with VECSTATIN ABC Elite kit (Vector Labs, Burlingame, CA) with DAB (Vector Labs) as a chromagenic substrate with hematoxylin counter-staining.

Images were acquired using a Nikon Labphot microscope with Nikon Plan Fluor 20 Ph3DL (numerical aperture: 0.75) objective (Nikon Instruments, Inc, NY) and Spot v.3.5.9 (Diagnostic Instruments Sterling Heights, MI) digital imaging system. The images from the tissue stained for mBCL2 were opened in Photoshop (Adobe Systems Inc. San Jose, CA), and staining intensity was determined by luminosity of the image with no alteration to the original image.

### Statistical Analysis

Results are presented as the means ± SE of the mean. Statistical analysis was performed by two-tailed t-test or Mann-Whitney test using GraphPad Prism 4 software. Differences associated with probability values of p<0.05 were considered statistically significant.

## Results

### Pretreatment with rhBCL2 Protein Reduces I/R Injury of Hind Limb Skeletal and Cardiac Muscle

**Figure 1:**
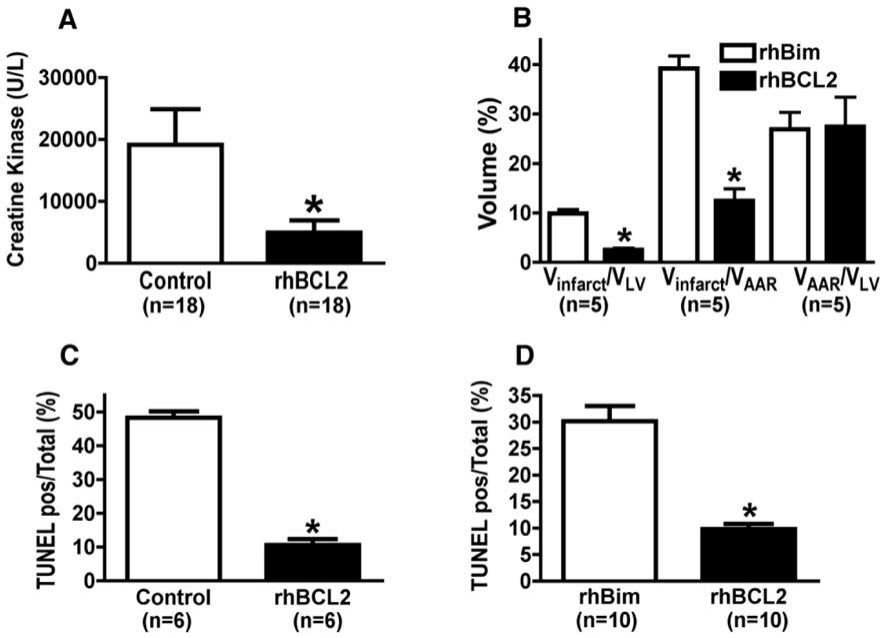
rhBCL2 protects hind limb skeletal muscle and cardiac muscle from I/R injury and apoptosis. (A) Creatine kinase (CK) levels in plasma were determined in mice treated ip with rhBCL2 protein (1 µg/mouse) or rhUbiquitin (0.5 µg/mouse) or vehicle solution the day prior to the hind limb I/R. Ischemia was produced by cross-clamping of the aorta for 90 min followed by 3 hrs of reperfusion. There were no differences in plasma CK levels between the rhUbiquitin-treated and vehicle-treated mice, and results from these treatments were combined. Muscle injury following I/R was significantly reduced by pretreatment with rhBCL2 (*p<0.05 rhBCL2 vs. control). (B) Mice were subjected to 60 minutes of LAD coronary artery occlusion and 2 hours of reperfusion and then evaluated for infarct size. Volume of area at risk (VAAR), left ventricle volume (VLV), and infarct volume (Vinfarct) were determined in mice pretreated the day prior to cardiac I/R with rhBCL2 (1 µg/mouse ip) or rhBim (1 µg/mouse ip). Treatment with rhBCL2 significantly reduced Vinfarct compared to treatment with rhBim (*p<0.001). (C) The number of TUNEL-positive nuclei in skeletal muscle of the rhBCL2-pretreated mice, expressed as a percent of the total number of nuclei, was reduced compared with control mice (*p<0.001). (D) The number of TUNEL-positive nuclei in cardiomyocytes, expressed as a percent of the total number of nuclei, was reduced in rhBCL2-pretreated mice compared with rhBim-pretreated mice (*p<0.001).

C57BL/6 mice were given ip injections of rhBCL2 protein (1 µg/mouse or ∼50 ng/g) or equimole amounts of rhUbiquitin protein or rhBim, a pro-apoptotic BCL2 family protein, or the vehicle solution on the day prior to I/R. Ischemia was produced by cross clamping of the aorta for 90 minutes. Plasma CK was measured as an indicator of skeletal muscle injury following 3 hours of reperfusion [10]. There was no difference in the plasma CK concentration between the rhUbiquitin-pretreated and vehicle control-pretreated mice following I/R, and these data were combined. In additional experiments, there was no difference between pretreatment with rhUbiquitin and two pro-apoptotic members of the BCL2 family, rhBim and rhBax (data not shown). Pretreatment with rhBCL2 resulted in significantly lower plasma CK concentration compared with the combined control treatment (Figure 1A).

In separate experiments, mice were subjected to 60 minutes of LAD coronary artery occlusion followed by 2 hours of reperfusion, and then evaluated for infarct size. C57BL/6 mice were given rhBCL2 or rhBim by ip injection on the day prior to I/R. There was no difference in volume of area at risk (VAAR) relative to left ventricular volume (VLV) between the groups (Figure 1B). The volume of infarct (Vinfarct) relative to the VLV in rhBCL2-pretreated mice was significantly less than in rhBim-treated mice (Figure 1B). Mean infarct size, expressed as a percent of the VAAR, was significantly less in the rhBCL2-pretreated mice compared with rhBim-pretreated mice (Figure 1B).

The marked reduction in I/R injury of skeletal muscle or cardiac muscle observed following pretreatment with rhBCL2 (as shown in Figure 1A and 1B) was associated with decreased apoptosis as detected by TUNEL staining (Figure 1C–D).

### The BCL2 Homologue rhBCL2A1 Also Reduces I/R Injury in Hind Limb Skeletal Muscle of Mice and Rats

**Figure 2:**
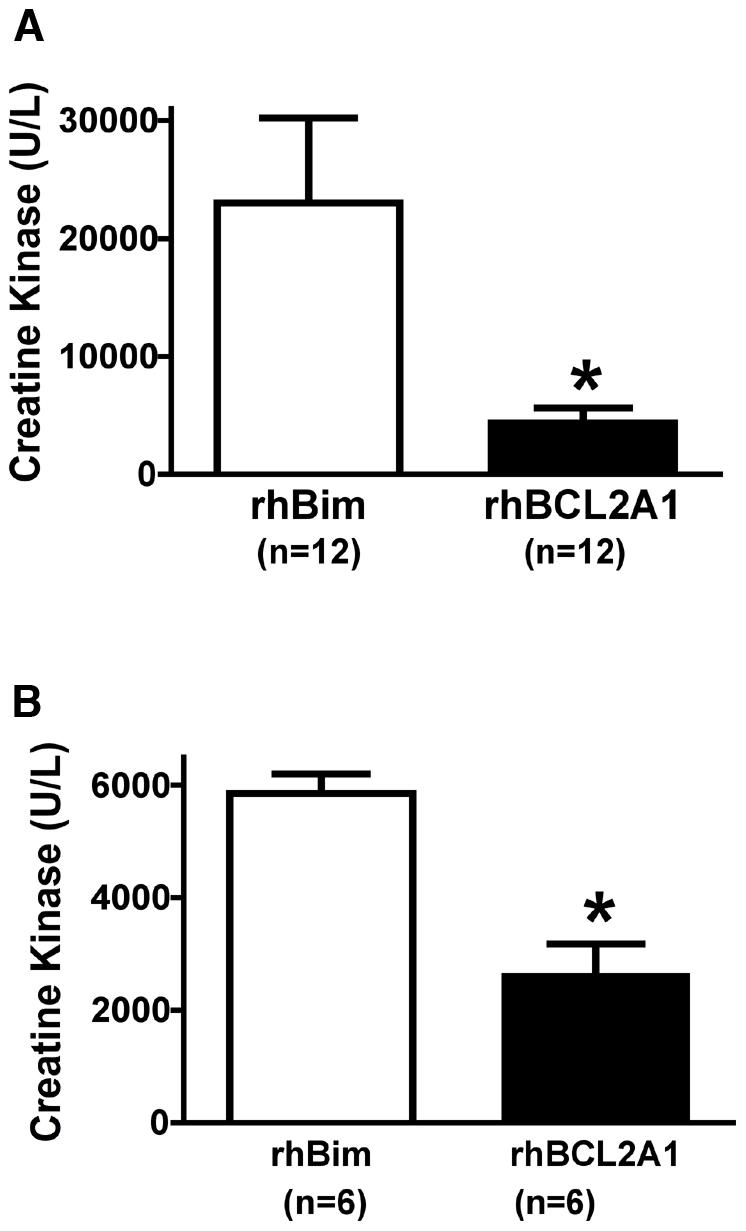
Pretreatment with rhBCL2A1 protects hind limb skeletal muscle from I/R injury. (A) Plasma CK levels were determined in mice pretreated with rhBCL2A1 protein (1 µg/mouse ip) or rhBim (1 µg/mouse ip) the day prior to hind limb I/R by aorta cross-clamping. Plasma CK levels were significantly decreased in the rhBCL2A1-pretreated mice (*p<0.05). (B) Plasma CK levels were measured in rats pretreated with rhBCL2A1 protein (10 µg/rat ip) or rhBim (10 µg/rat ip) the day prior to hind limb I/R by tourniquet induced for 90 minutes of ischemia, followed by 3 hours of reperfusion. Plasma CK levels were significantly reduced in the rhBCL2A1-pretreated rats (*p<0.05).

In order to determine whether the protection against I/R injury was restricted to BCL2, we examined the effect of rhBCL2A1, an anti-apoptotic BCL2 family member [11]–[13]. Mice were pretreated with rhBCL2A1 or rhBim on the day prior to I/R. Hind limb ischemia was induced by aorta cross clamping for 90 minutes, and plasma CK levels were measured 3 hours after reperfusion. As shown in Figure 2A, the CK level was significantly less in the rhBCL2A1-pretreated mice compared with the rhBim-pretreated mice. These results demonstrate that another anti-apoptotic protein of the BCL2 family has a similar cytoprotective function when administered extracellularly.

In additional experiments, rats were pretreated with rhBCL2A1 in order to determine whether the protection noted in the mouse following I/R injury was species-dependent. Plasma CK levels in rats pretreated with rhBCL2A1 the day prior to tourniquet I/R of hind limb were significantly less than CK levels in rats pretreated with rhBim (Figure 2B).

### BH4- and BH4-Like Peptides Protect Hind Limb Skeletal Muscle from I/R Injury

The two anti-apoptotic members of the BCL2 family, rhBCL2 and rhBCL2A1, were protective against I/R injury when given extracellularly, whereas the pro-apoptotic members, rhBim and rhBax, were not. BCL2 contains an N-terminal domain, designated BH4, which is present in several other anti-apoptotic family members, but is not present in pro-apoptotic members such as Bim or Bax. A BH4-like domain has been identified in the first alpha-helix of BCL2A1 by molecular modeling [6]. Therefore, we examined the role of the BH4 domain in the extracellular cytoprotective function of BCL2.

**Figure 3:**
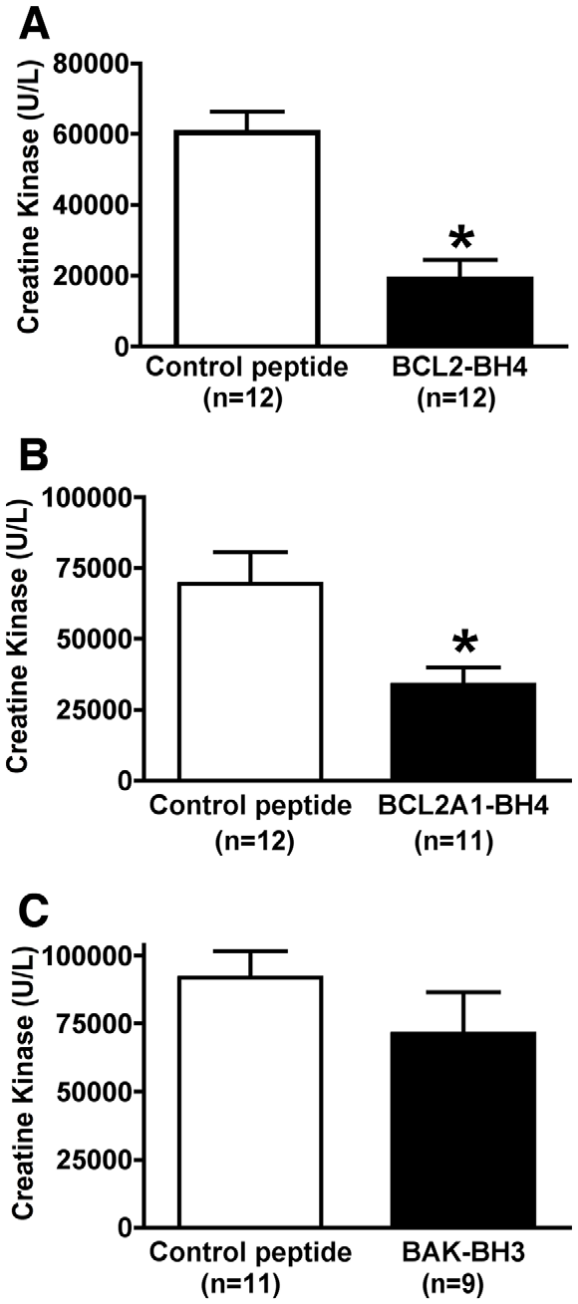
Peptides from BH4 domains of hBCL2 and hBCL2A1 protect skeletal muscle From I/R injury. Mice were pretreated with (A) synthetic BH4-BCL2 peptide (10 µg/mouse), (B) BH4-BCL2A1 peptide (10 µg/mouse), (C) BH3-Bak peptide (10 µg/mouse), or control scrambled peptide (10 µg/mouse) given ip on the day prior to hind limb I/R induced by tourniquet for 90 minutes of ischemia, followed by 3 hours of reperfusion. Plasma CK levels were significantly less in mice pretreated with hBCL2-BH4 or hBCL2A1-BH4 compared to mice pretreated with a scrambled peptide (*p<0.05).

When the BH4 domain of BCL2 was deleted (BCL2-delBH4), pretreatment with this mutant BCL2 protein failed to protect against I/R injury of hind limb skeletal muscle (plasma CK levels following aorta cross-clamp and release were 12161 ± 4578 after pretreatment with rhBim vs. 16369 ± 8109 with rhBCL2-delBH4; p = 0.66), consistent with a crucial role of BH4 domain in the cytoprotective activity of extracellular rhBCL2. Peptides from the BH4 domain of rhBCL2 (hBCL2-BH4) or the BH4-like domain of rhBCL2A1 (hBCL2A1-BH4) were synthetized and tested in the I/R experiments. Synthetic hBCL2-BH4 and hBCL2A1-BH4 peptides were both effective in reducing I/R, whereas a peptide from the BH3 domain of Bak, a pro-apoptotic BCL2 family member, was not (Figure 3).

### Treatment with rhBCL2A1 Protein at the Time of or Following Reperfusion Protects Skeletal Muscle against I/R Injury

**Figure 4:**
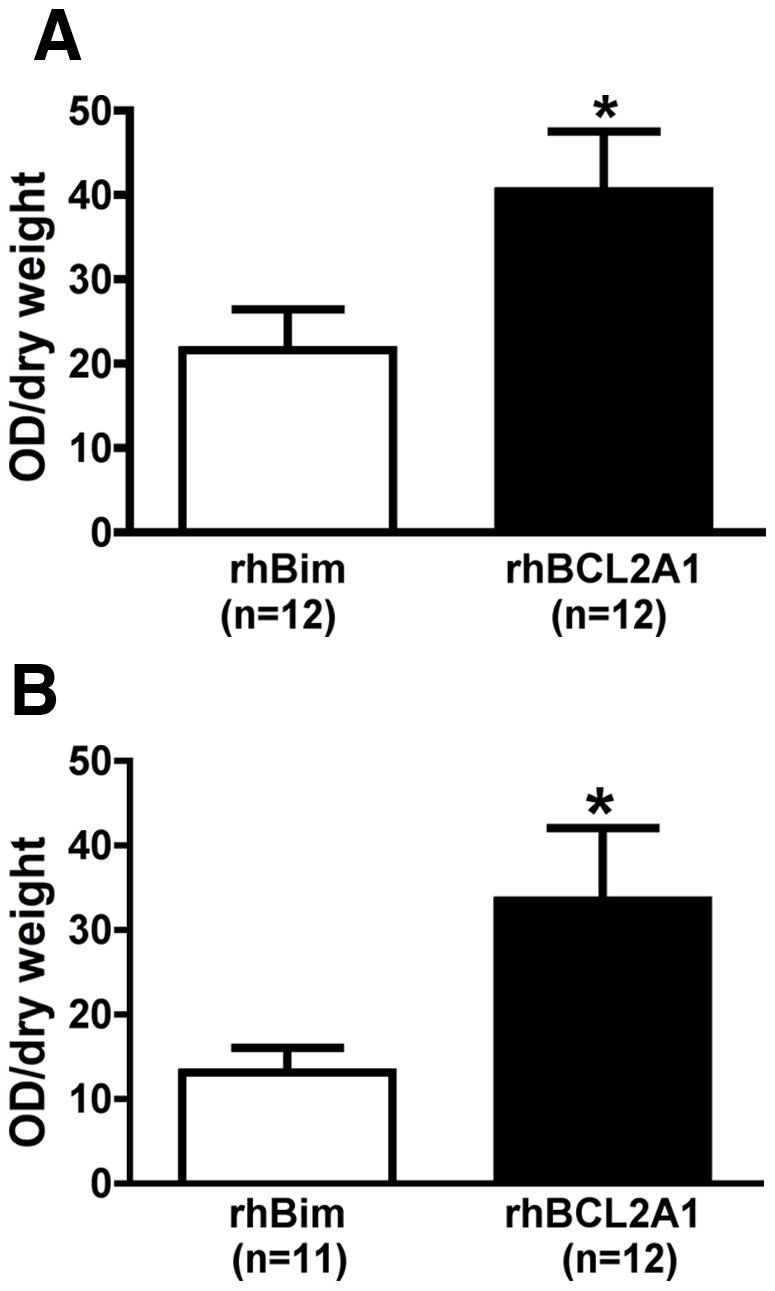
Treatment with rhBCL2A1 at the time of or following reperfusion protects hind limb skeletal muscle from I/R injury. Mice were subjected to tourniquet ischemia for 90 minutes, and then treated with subcutaneous rhBCL2A1 or rhBim (1 µg/mouse) at the time of reperfusion (A) or 4 hours after the start of reperfusion (B). Tissue viability was measured the following day by MTT assay (see methods section). Tissue viability was significantly greater in the rhBCL2A1-treated mice compared with the rhBim-treated mice (*p<0.05).

Mice were subjected to I/R of hind limb by tourniquet, and tissue viability was measured at 24 hours after reperfusion using MTT [9]. Viability of tissue was measured by the ability of mitochondria to reduce MTT to a formazan salt that is insoluble in water. The formazan crystals are soluble in isopropanol and are extracted from tissue by incubating the tissue in isopropanol. Thus, live tissue in this assay will have increased optical density relative to dead tissue. Optical density of the tissue normalized to tissue dry weight from mice treated with rhBCL2A1 protein given subcutaneously at the time of reperfusion is shown in Figure 4A. Clearly, the viability of tissue from mice treated with rhBCL2A1 protein was greater than that of mice treated with the control protein rhBim. These experiments demonstrated that rhBCL2A1 protein was effective when administered at the time of reperfusion. Notably, subcutaneous administration of rhBCL2A1 as late as 4 hours following reperfusion resulted in significantly greater tissue viability at 24 hours after reperfusion compared with mice treated with rhBim (Fig. 4B). Remarkably, treatment with as little as 100 ng per mouse of rhBCL2A1 (∼5 µg/kg or ∼4 picomoles) was effective in reducing tissue injury (Fig. S1A) and treatment was effective even when given up 72 hours prior to I/R (Fig. S1B).

These experiments demonstrate that rhBCL2A1 protein is effective in attenuating I/R injury at cytokine-like doses given prior to, at the time of, or following reperfusion.

### Treatment with rhBCL2A1 Protein at the Time of Reperfusion Reduces Myocardial Infarct Size at 24 Hours

**Figure 5:**
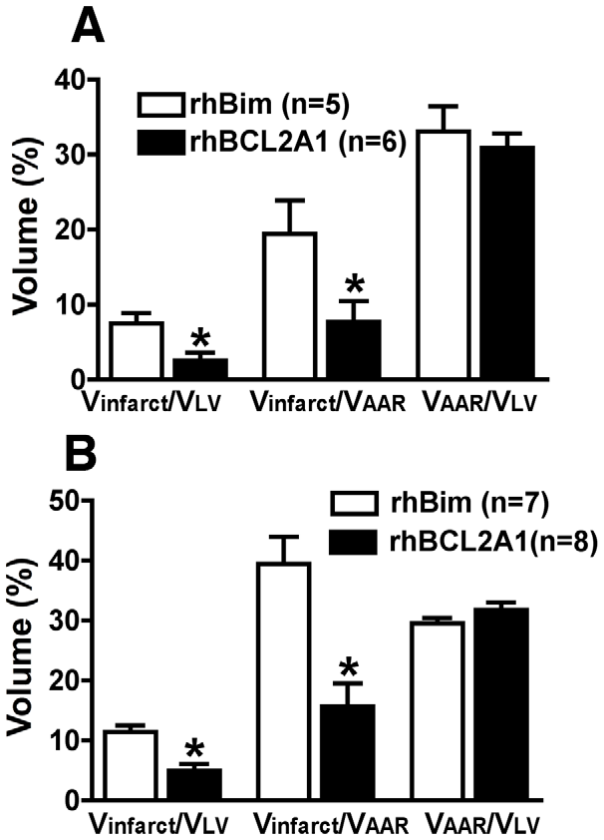
Treatment with rhBCL2A1 at the time of reperfusion reduces myocardial infarct size after I/R. Mice were subjected to LAD artery occlusion for 1 (A) or 2 (B) hours, and treated with subcutaneous rhBCL2A1 (1 µg/mouse) or rhBim (1 µg/mouse) at the time of reperfusion. Infarct size was determined after 24 hours of reperfusion (see Methods). Treatment with rhBCL2A1 at the time of reperfusion reduced infarct size at 24 hours of reperfusion (*p<0.05).

C57BL/6 mice were subjected to 60 minutes of LAD coronary artery occlusion with treatment at the end of ischemia by subcutaneous injection of rhBCL2A1 or rhBim. Reperfusion was then continued for 24 hours before evaluation of infarct size. There was no difference in the volume at risk (VAR) relative to left ventricle (LV) between the groups (Figure 5A). The infarct size relative to the LV in rhBCL2A1-treated mice was significantly less than in rhBim-treated mice. Mean infarct size, expressed as a percentage of the VAR, was significantly less in the rhBCL2A1-treated mice compared with rhBim-treated mice.

In additional experiments, C57BL/6 mice were subjected to more severe ischemia by occluding the LAD coronary artery for 120 minutes with treatment at the end of ischemia at the time of reperfusion by subcutaneous injection of rhBCL2A1 or rhBim. Reperfusion was then continued for 24 hours before evaluation of infarct size. There was no difference in VAAR relative to VLV between the groups (Figure 5B). The infarct volume (Vinfarct) relative to VLV in rhBCL2A1-treated mice was significantly less than in rhBim-treated mice. Infarct volume (Vinfarct), expressed as a percentage of the VAAR, was significantly less in the rhBCL2A1-treated mice compared with rhBim-treated mice.

### TLR2-MyD88 Signaling Is Required for rhBCL2A1 to Protect Hind Limb Skeletal Muscle against I/R Injury

Since BCL2 and BCL2A1 do not have significant primary homology in the BH4 region, we reasoned that their effects *in vivo* may involve a pattern recogniztion receptor. Since toll-like receptors (TLRs), particularly TLR2 and TLR4, have been demonstrated to recognize endogenous ligands, including DAMPs, as well as microbial components [1], [3], [14], we examined TLR2 and TLR4 signaling for their involvement in the protection afforded by administration of rhBCL2 or rhBCL2A1.

**Figure 6:**
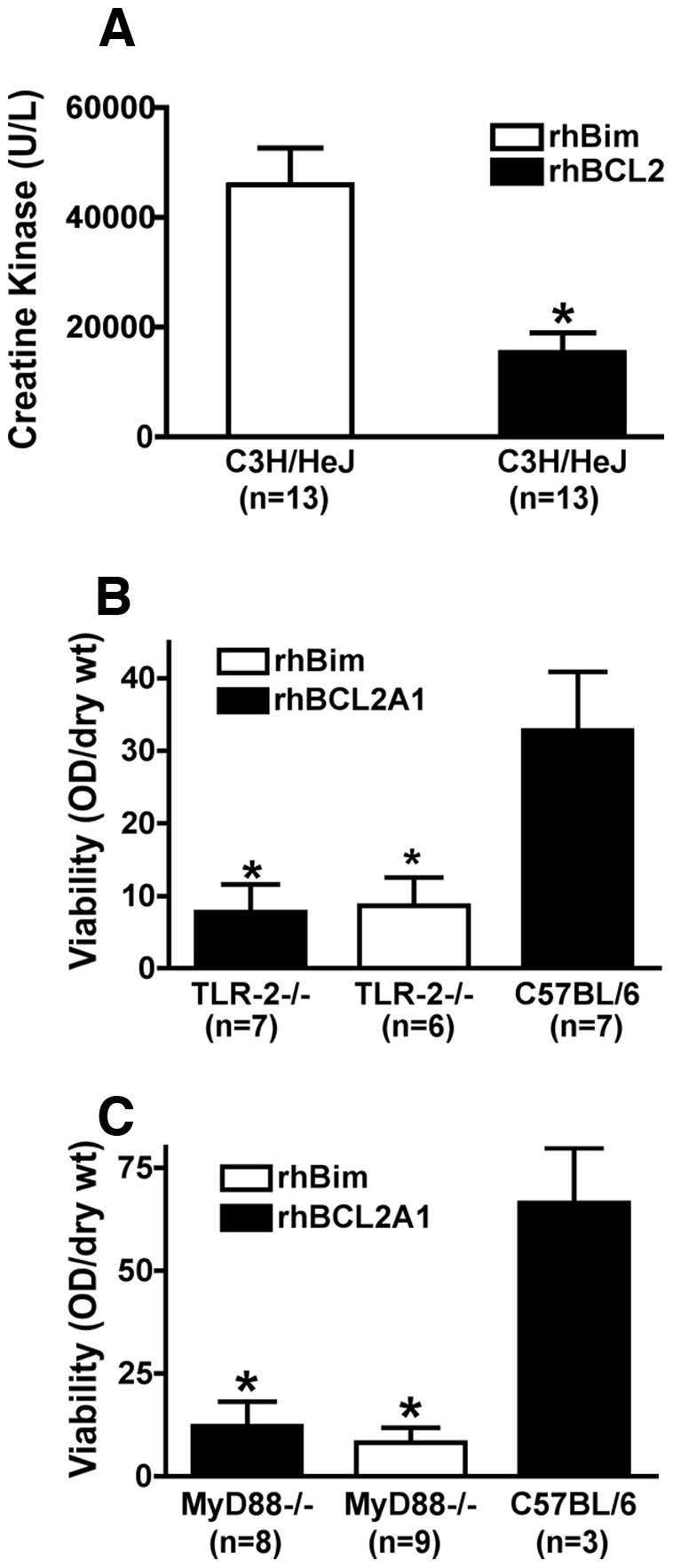
Protection of hind limb skeletal muscle from I/R Injury requires TLR2-MyD88 but not TLR4 signaling. (A) C3H/HeJ (TLR4 mutant) mice were pretreated with rhBCL2 (1 µg/mouse ip) or rhBim (1 µg/mouse ip) on the day prior to I/R. Mice were subjected to 90 minutes of hind limb ischemia by tourniquet, followed by 3 hours of reperfusion. CK levels in the plasma from mice were reduced by pretreatment with rhBCL2 (*p<0.05). (B)(C) C57BL/6, TLR2^−/−^ or MyD88^−/−^ mice were pretreated with rhBCL2A1 or rhBim given subcutaneously (1 µg/mouse) at the time of reperfusion. Tissue viability was assessed after 90 minutes of hind limb ischemia by tourniquet and 24 hours of reperfusion. Tissue viability in TLR-2^−/−^ (B) and MyD88^−/−^ (C) mice treated with rhBCL2A1 or rhBim was reduced compared to C57BL/6 mice treated with rhBCL2A1 (*p<0.05).

C3H/HeJ mice with a mutation in TLR4 that prevents signaling [15] were pretreated the day prior to tourniquet I/R with rhBCL2 or rhBim. As shown in Figure 6A, plasma CK levels were significantly lower in rhBCL2-treated mice than in mice treated with rhBim.

TLR2-null (TLR2^−/−^) mice (on a C57BL/6 background) were subjected to tourniquet I/R, and treated with either rhBCL2A1 or rhBim given subcutaneously at the time of reperfusion. Tissue viability was measured at 24 hours after reperfusion using the MTT assay [9]. These mice were compared with C57BL/6 mice treated similarly with rhBCL2A1 or rhBim. Optical density of the tissue normalized to tissue dry weight from mice treated at the time of reperfusion is shown in Figure 6B. Clearly, the TLR2^−/−^ mice were not protected by rhBCL2A1.

Since MyD88-independent TLR2 signaling has been described [16], we also examined MyD88-null (MyD88^−/−^) mice. MyD88^−/−^ mice were subjected to tourniquet I/R treated with rhBCL2A1 or rhBim at the time of reperfusion. Tissue viability was measured at 24 hours after reperfusion using the MTT assay. In concurrent experiments, C57BL/6 mice were treated similarly with rhBCL2A1 or rhBim. Optical density of the tissue normalized to tissue dry weight from mice treated at the time of reperfusion is shown in Figure 6C. MyD88^−/−^ mice were not protected by treatment rhBCL2A1, indicating that the canonical TLR2-MyD88 pathway was required.

### Treatment with Exogenous rhBCL2 Protein Increases Expression of Endogenous Mouse BCL2 Protein in Skeletal and Cardiac Muscle Following I/R

**Figure 7:**
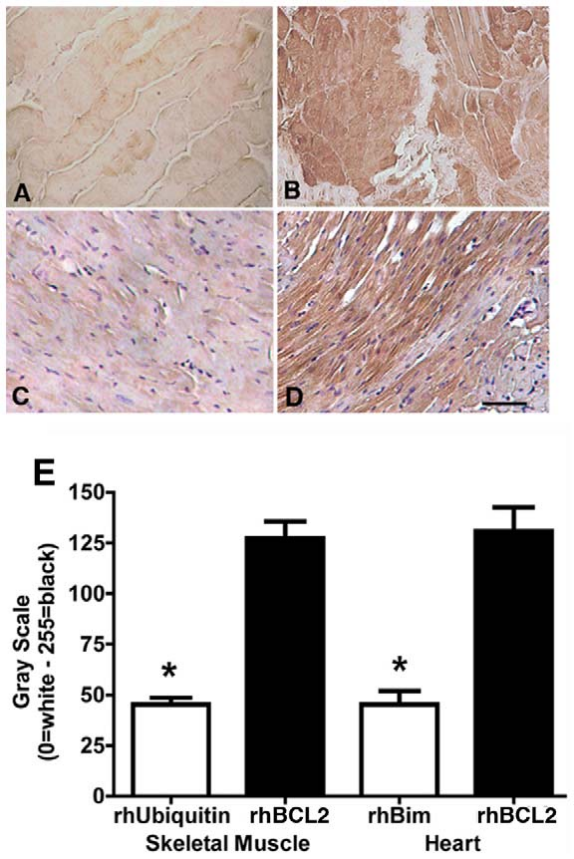
Expression of endogenous mouse BCL2 protein in cardiomyocytes and hind limb skeletal muscle cells is increased after I/R in mice treated with rhBCL2. Expression of endogenous mouse (m) BCL2 protein in tissue subjected to I/R was determined by staining with a mouse-specific anti-BCL2 antibody. (A, B) Mice were pretreated ip with (A) rhUbiquitin (1 µg/mouse) or (B) rhBCL2 (1 µg/mouse) on the day prior to hind limb I/R by tourniquet induced by 90 minutes of ischemia followed by 3 hours of reperfusion. (C, D) Mice were pretreated with (C) rhBCL2 (1 µg/mouse) or (D) rhBim (1 µg/mouse) on the day prior to cardiac I/R induced by 60 minutes of ischemia and 2 hours of reperfusion. Expression of endogenous mBCL2 protein was increased in skeletal muscle and cardiomyocytes after I/R in the rhBCL2-pretreated mice compared to mice pretreated with rhUbiquitin or rhBim. The scale bar represents 100 µm. (E) Quantitative morphometric analysis on the skeletal muscle and heart from rhBCL2-pretreated mice, respectively. Skeletal muscle and heart from rhBCL2-pretreated mice showed increased expression of endogenous mBCL2 protein compared with tissue from mice pretreated with control proteins (*p<0.05).

Treatment with rhBCL2 or rhBCL2A1 reduced apoptosis of skeletal and cardiac muscle following I/R, suggesting that the protein might protect by inducing expression of an endogenous anti-apoptotic protein(s). Consequently, we examined expression of endogenous mouse (m)BCL2 protein in muscle cells following I/R using an antibody specific for mouse BCL2. As shown in Figure 7A there was minimal expression of mBCL2 protein after I/R injury in skeletal muscle of control mice. However, tissue following I/R from rhBCL2-treated mice showed a significant increase in mBCL2 protein expression compared with control mice (Figs. 7B and 7E). Likewise, mBCL2 protein expression in the hearts of control mice exhibited minimal expression of mBCL2 protein following I/R (Figure 7C), whereas mBCL2 protein was significantly increased in mice treated with rhBCL2 following I/R injury (Figures 7D and 7E). Pretreatment with rhBCL2A1 also increased expression of endogenous mBCL2 protein in skeletal muscle cells following I/R (data not shown).

### rhBCL2A1 Does Not Directly Activate TLR2 or Reduce Apoptosis *In Vitro*

We attempted to replicate the *in vivo* activities of BCL2A1 using *in vitro* models. rhBCL2A1 (up to 10 µg/ml) did not directly activate TLR2 receptors as determined by IL-8 production in HEK cells transfected with TLR2/6 (Fig. S2) or by nuclear factor-kappa B (NF-κB)-dependent activation in THP1 macrophages (Methods S1, Fig. S3). Also, the response of THP-1 macrophage cells to several TLR2 ligands was unaffected by pretreatment with rhBCL2A1 (Methods S1, Fig. S4).

We determined effect of rhBCL2A1 on the induction of apoptosis *in vitro* in number of other cell types (e.g., endothelial, fibroblasts, lymphocytes) challenged with a variety of pro-apoptotic stimuli (e.g., Fas, H_2_0_2_) (unpublished observations). Results in HEK TLR2/6 transfectants challenged with etoposide or the 32D myeloid cell line subjected to growth factor deprivation are shown in Figures S5 and S6, respectively.

## Discussion

The results of experiments described in this report demonstrate a previously unreported extracellular cytoprotective function for the intracellular anti-apoptotic proteins, BCL2 and BCL2A1. Thus, BCL2 and BCL2A1 and BH4-domain peptides join a growing list of intracellular DAMPs (reviewed in [2], [3]).

The BCL2 family contains both pro- and anti-apoptotic proteins and their intracellular actions regulating apoptosis have been extensively investigated (reviewed in [4]). There are two sub-families of pro-apoptotic proteins with one sub-family consisting of multiple domain proteins (Bax, Bak, Bod) and the other sub-family consisting of BH3 domain-only proteins (e.g., Bim, Bad, and Bik)(reviewed in [17]). The anti-apoptotic members of the BCL2 family have multiple domains, and most, but not all, have a primary structure designated as the BH4 domain (reviewed in [17]). The secondary structure of BCL2L1 (Bcl-xL) [18] and a chimeric BCL2L1/BCL2 protein [19] have been determined and shown to contain multiple alpha-helices with the N-terminal alpha-helix containing the BH4 domain. The BH4 domain has been shown to be necessary for the anti-apoptotic function of intracellular BCL2 [20]. Although the primary structure of BCL2A1 does not reveal a BH4 domain [21], using molecular modeling Zhang *et al* [6] showed that there is a structural homolog of the BH4 domain in the first alpha-helix of BCL2A1. In contrast, pro-apoptotic BCL2 family members such Bax or Bim do not have either the primary or secondary structure of a BH4 or BH4-like domain [21].

In experiments reported here, pretreatment with synthetic peptides from the BH4 domain of BCL2 and the BH4-like domain of BCL2A1 conferred protection, whereas deletion of the BH4 domain from BCL2 abrogated protection against I/R injury. These results suggest that the secondary structure of the BH4 or BH4-like domain was critical to the extracellular cytoprotective activity. There are several reports showing that a construct of the BH4 peptide from Bcl-Xl conjugated to the cell penetrating HIV-TAT peptide sequence blocked apoptotic cell death *in vitro* [22]–[25] and *in vivo* [25]–[27]. However, our studies are the first to show an extracellular function *in vivo* for a BCL2-BH4 or BCL2A1-BH4 peptide without a protein transduction domain.

The predicted structural homology between the BH4 domain of BCL2 and the first alpha-helix of BCL2A1 (i.e., BH4-like domain) [6], suggested that the protective effect of BCL2, BCL2A1 and their respective BH4 peptides may involve a pattern recognition receptor. Since toll-like receptors have been shown to recognize a broad spectrum of microbial products as well as DAMPs [2], [3], we investigated their involvement in the reduction of skeletal muscle I/R injury provided by rhBCL2 or rhBCL2A1. The protection observed against I/R injury in the C3H/HeJ mice indicated that the protective effect of rhBCL2 was not dependent upon TLR4 signaling. In contrast, the failure of rhBCL2A1 to protect the TLR2^−/−^ or MyD88^−/−^ mice demonstrates that TLR2 signaling through a MyD88-dependent pathway is required for in the reduction in injury. Of interest, TLR2 has been shown to be a primary sensor of necrotic cells [28], [29]. Although implicated in pro-inflammatory responses after I/R (reviewed in [30]), TLR2 signaling has also been shown to be protective in some I/R models [31]–[33]. In contrast to reports in other I/R models (e.g., [30]), neither the TLR2^−/−^ nor the MyD88^−/−^ mice exhibited increased hind limb skeletal muscle injury compared to controls. This may reflect the severity of our model with a prolonged ischemia time that produces an injury that is not dependent upon leukocytes [7].

The skeletal muscle cell injury produced by extended I/R was previously shown to have a prominent component of apoptosis as measured by TUNEL staining, and active caspase-3 staining and by reduction of injury by a broad-spectrum caspase inhibitor [7]. Mice treated with rhBCL2 or rhBCL2A1 protein exhibited significantly less apoptosis in affected tissue following I/R. Previous investigations have shown over-expression of BCL2 in critical target cells reduces organ injury following I/R (e.g., [34]–[40]). Notably, mice treated with rhBCL2 or rhBCL2A1 showed increased endogenous mBCL2 protein expression in heart or skeletal muscle following I/R. These results suggest that one mechanism by which exogenously administered rhBCL2 or rhBCL2A1 protects tissue against I/R-induced apoptosis is by augmenting increased expression of endogenous mouse BCL2. Of note, we have not been able to detect a direct effect of rhBCL2 or rhBCL2A1 on apoptosis *in vitro* in HEK TLR2 transfectants challenged with etoposide or in a myeloid cell line subjected to growth factor deprivation (Figs. S5 and S6) or in a number of other cell types (e.g., endothelial, fibroblasts, lymphocytes) challenged with a variety of pro-apoptotic stimuli (e.g., Fas, H_2_0_2_) (unpublished observations). Given the lack of a detectable direct effect *in vitro*, the TLR2 dependence *in vivo*, and the efficacy with picomole quantities of protein, it seems unlikely that the potent anti-apoptotic effect observed *in vivo* reflects uptake of protein and inhibition of apoptosis by the usual mechanisms ascribed to BCL2. As discussed below, it is more likely that the anti-apoptotic activity of extracellular BCL2 or BCL2A1 *in vivo* in the setting of I/R involves a novel pathway, perhaps akin to preconditioning induced by other DAMPs or by TLR ligands [41].

The cellular and molecular pathway(s) from rhBCL2A1 or rhBCL2 ⇒ TLR2-MyD88 ⇒ cytoprotection *in vivo* remains to be elucidated. Although TLR2-MyD88 signaling is required *in vivo*, at concentrations relevant to the *in vivo* studies rhBCL2A1 does not directly activate TLR2 *in vitro*. There are several potential explanations for this discrepancy between the *in vivo* and *in vitro* results. First, there may be an intermediate effector cell *in vivo* that then elaborates an endogenous TLR2 ligand(s) in response to stimulation by rhBCL2A1 or rhBCL2. Second, the BCL2A1-triggered cytoprotective pathway may be present only in a subset of TLR2-expressing cells. In this regard, inflammatory monocytes, but not bone marrow-derived macrophages or dendritic cells, were recently shown to produce type 1 interferon via TLR2-MyD88 signaling in response to viral but not bacterial ligands [42]. Notably, induction of type 1 interferon in inflammatory monocytes required internalization of TLR2. Third, BCL2, BCL2A1, or BH4-domain peptides may require a co-factor for activity *in vivo*. For example, it has recently been shown that recombinant high mobility box 1 protein (HMGB1), a prototypic alarmin or DAMP, lacks significant activity *in vitro* when it is highly purified [43]–[45], but acquires activity *in vitro* upon binding to some TLR ligands [45] or cytokines [44]. (Interestingly, although first characterized by its pro-inflammatory activities, preconditioning with low-doses of HMGB1 confers protection in liver I/R injury [46].) Finally, TLR2 heterodimerizes with TLR1 and TLR6, and TLR2 signaling is modulated by other non-TLR receptors such as dectin-1 [47], asioloGM1 [48], and CXCR4 [49]. It is possible that BCL2 or BCL2A1 protein engages a co-receptor and that co-operative signaling by this receptor and TLR2 (engaged by endogenous ligands generated with tissue injury) promotes cytoprotection. Such a scenario has been proposed for the cardioprotection observed following treatment with β-glucan [50], a dectin-1 ligand.

In summary, we report that severe I/R injury to skeletal or cardiac muscle was reduced by very low doses of exogenously administered rhBCL2 protein or rhBCL2A1 protein or BH4 peptides. These results demonstrate a novel, cytokine-like function for extracellular BCL2 and BCL2A1 proteins as cytoprotective DAMPs. In this regard, in preliminary studies we have detected BCL2 in supernatant medium of lysed cells (Fig. S7). More importantly, soluble BCL2 protein has been detected in serum in a variety of disorders [51]–[55] and cerebrospinal fluid in traumatic brain injury [56], [57]. Notably, in children following head trauma increased levels of soluble BCL2 protein in spinal fluid correlated with improved survival [57]. Since a BH4-domain containing peptide is generated by caspase cleavage of BCL2, it is likely that BH4-peptides are also released extracellularly [58]. We further speculate that the BH4-containing proteins or peptides interact with cells in target tissue or, perhaps more likely, with an intermediate effector cell to prime for a cytoprotective response in stressed tissue. The inability of rhBCL2A1 to protect the TLR2^−/−^ or MyD88^−/−^ mice against I/R injury of skeletal muscle indicates a requisite role for TLR2-MyD88 signaling for cytoprotection *in vivo*.

Finally, the protective effect of the extracellularly administered BH4-domain proteins is not limited to I/R. In other studies, we found that treatment with rhBCL2 or rhBCL2A1 reduced apoptosis in target organs and improved survival in a sepsis model (manuscript in preparation). In preliminary studies, treatment with a single dose of rhBCL2A1 also significantly attenuated cardiomyocyte apoptosis, heart weight gain, and fibrosis in a heart failure model (Methods S1; Figs. S8 and S9). Thus, extracellular administration of BH4-containing proteins or peptides may offer a new approach to therapy of human diseases associated with cell death.

## Supporting Information

Methods S1 (0.04 MB DOC)

Figure S1 Protection against I/R injury by rhBCL2A1 is dose-dependent and persists for up to 72 hours. (A) Mice were subjected to tourniquet ischemia for 90 minutes, and treated with subcutaneous rhBCL2A1 (1 Î¼g/mouse) at the time of reperfusion. Tissue viability, measured the following day by MTT assay, was increased by treatment with rhBCL2A1 in dose-dependent manner (*p<0.01 vs. saline, **p<0.05 vs. saline). (B) Mice were treated with subcutaneous rhBCL2A1 or rhBim (1 Î¼g/mouse) at 72 hrs prior to 90 minutes of ischemia followed by 24 hours of reperfusion. Tissue viability as measured by MTT assay was significantly greater in the rhBCL2A1-treated mice compared with the rhBim-treated mice (*p<0.05).
(7.23 MB TIF)

Figure S2 rhBCL2A1 does not induce release of IL-8 from TLR2/6-transfected HEK cells. Wild-type human embryonic kidney (HEK) cells and HEK-293 cells stably transfected with TLR2 and TLR6 (HEK TLR2/6) were obtained from InvivoGen (San Diego, CA). The HEK cells were incubated with BCL2A1 protein (10 Î¼g/ml) or with the TLR2/6 ligand FSL-1 (100 ng/ml; InvivoGen) for six hours. Supernatant medium was sampled for IL-8 determination by ELISA (eBioscience).
(0.14 MB TIF)

Figure S3 rhBCL2A1 does not induce NF-kappa B activation in THP-1 macrophage-like cells. CControl medium or medium containing BCL2A1 protein (0.1, 0.5 or 1 Î¼g/ml) or E. coli lipopolysaccharide (LPS) (0.1, 1 or 10 ng/ml) was incubated with THP1-Blue (TM) cells for 24 h. Samples were removed for analysis of NF-kappa B production by secreted embryonic alkaline phosphatase (SEAP) as described below in Methods. Gray bar, control medium; open bars, BCL2A1 protein; black bars, LPS. Results represent means Â± SD of 6 replicates.
(0.21 MB TIF)

Figure S4 BCL2A1 does not alter NF-kappa B activation in response to other TLR2 ligands. Control medium or BCL2A1 protein (100 ng/ml) was placed on THP1-Blue (TM) cells followed by the addition of Pam3CSK4 (100 ng/ml), zymosan (25 Î¼g/ml), or FSL-1 (10 or 100 ng/ml). Samples were removed to Quanti-Blue (TM) for measurement of SEAP production as described below. Ordinate: Quanti-Blue (TM), net absorbance. Open bars, control medium; black bars, BCL2A1 protein.
(0.15 MB TIF)

Figure S5 BCL2A1 protein or TAT-BCL2L1-BH4 peptide does not protect HEK cells from apoptosis. Wild-type (WT) or TLR2/6-transfected HEK-293 cells were treated overnight in complete medium alone or medium with (A) BCL2A1 protein (300 ng/ml) or (B) TAT-BCL2L1-BH4 (Bcl2-Xl) peptide (1 Î¼g/ml). TAT-BCL2L1 consists of the BH4 domain of BCL2L1 (aa 4–23) linked to a protein transduction domain peptide from HIV-TAT (aa 48–57) (EMD Chemicals, Inc., USA). After removal of medium, cells were then treated with etoposide (20 Î¼M) in complete medium for 23 hrs and assessed for viability with Alamar Blue. Open bars, control medium; black bars, (A) BCL2A1 or (B) TAT-BCL2L1-BH4.
(0.19 MB TIF)

Figure S6 BCL2A1 does not prevent apoptosis induced by growth factor withdrawal. 332D myeloid cells are dependent upon IL-3. 32D cell were grown overnight in complete medium containing IL-3 with or without BCL2A1 (30 Î¼g/ml). The cells were then aliquoted to fresh medium with or without IL-3. Cells pretreated with BCL2A1 (A1) overnight were further divided to different concentrations of BCL2A1 (0 to 30 Î¼g/ml). After a 24 hr incubation viability was determined by Alamar Blue assay (Invitrogen).
(0.21 MB TIF)

Figure S7 Necrotic cells release BCL2. Jurkat cells, a human T-cell line, were lysed in one ml of complete lysis buffer or were suspended in one ml of PBS containing protease inhibitor cocktail (Sigma) and subjected to three cycles of freeze-thaw. Cell debris was removed by centrifugation at 600 g for 10 min and then 12,000 g for 20 min. Protein concentrations were determined and detergents and EDTA were then added to the freeze-thaw sample to mimic the lysed sample. hBCL2 protein in supernatants was measured by ELISA (R and D Systems), according to the manufacturer's directions.
(0.17 MB TIF)

Figure S8 rhBCL2A1 reduces cardiac hypertrophy and apoptosis induced by aorta-banding. (A) Bar graph shows the effect of rhBCL2A1 treatment on heart weight/body weight ratio at 3 weeks after aorta-banding procedure. At the time of aorta-banding mice were injected subcutaneously with rhBCLA1 or rhBim (1Î¼g/mouse). (*p<0.05) (B) Bar graph showing the effect of rhBCL2A1 on DNA strand breaks by TUNEL staining of heart at 3 weeks after aorta-banding procedure. At the time of aorta-banding mice were injected subcutaneously with rhBCLA1 or rhBim (1Î¼g/mouse). (*p<0.05)
(5.66 MB TIF)

Figure S9 hBCL2A1 reduces development of cardiac fibrosis following aorta-banding. Histological sections of hearts were stained with Sirius-red to highlight collagen. (A, B) show hearts from rhBim-treated animal, (C, D) from rhBCL2A1-treated animal. (A, C) show perivascular fibrosis, and (B, D) show myocardial fibrosis. (original magnification: ×40). (E, F) show quantification by morphometry of the Sirius-red staining area. (E) illustrates perivascular fibrosis index as measured by perivascular collagen normalized to the vascular luminal area. (F) illustrates myocardial interstitial fibrosis determined as percent of collagen area to myocardial area in microscopic field. (*p<0.05)
(5.43 MB TIF)
