# Supplementary material for: Extracellular BCL2 Proteins Are Danger-Associated Molecular Patterns That Reduce Tissue Damage in Murine Models of Ischemia-Reperfusion Injury
Source: PLoS One. 2010 Feb 8;5(2):e9103. doi: 10.1371/journal.pone.0009103 (PMC2816997; doi:10.1371/journal.pone.0009103)
Supplement: Methods S1 — (0.04 MB DOC) [file pone.0009103.s001.doc]

**SUPPORTING INFORMATION- METHODS**

*NF-κB activation in THP-1 macrophage cell line (Figs. S3 and S4):* THP1-Blue™ cells were obtained from InvivoGen, San Diego, CA. They are stably transfected with a reporter plasmid expressing a secreted embryonic alkaline phosphatase (SEAP) gene under the control of a promoter inducible by the transcription factors NF-κB and AP-1. Upon TLR stimulation, THP1-Blue™ cells induce the activation of NF-κB and AP-1 and subsequently the secretion of SEAP. The reporter protein is easily detectable and measurable when using Quanti-Blue™ (InvivoGen), a medium that turns purple/blue in the presence of SEAP. THP1-Blue™ cells were grown in RPMI 1640 containing GlutaMAX-1, sodium pyruvate and neomycin (Invitrogen, Grand Island, NY), sodium bicarbonate and HEPES (Hyclone, Logan, UT), 4.5% glucose (Sigma-Aldrich, St Louis, MO) and 10% fetal bovine serum (FBS) (Hyclone) with the addition of the following antibiotics: Zeocin, Blasticidin, and Normocin (InvivoGen). For experiments THP1-Blue™ cells were plated in medium with or without antibiotics with 10% FBS and with 50 ng/ml PMA. After two days they were washed twice daily with medium containing 2% FBS and fed medium containing 10% FBS. On the day of the experiment, they were washed with complete THP1-Blue™ medium (containing antibiotics) with 5% FBS and fed the same medium until reagents were prepared. The medium was removed and replaced with test reagents in complete THP1-Blue™ medium with 5% FBS. Samples were tested for SEAP production by adding them to plates containing Quanti-Blue™. Net absorbance was determined at 630 nm on an EL 340 microplate reader (Bio-Tek Instruments, Winsooski, VT) by subtracting absorbance of Quanti-Blue™ without sample.

*Heart failure Induced by aorta-banding (Figs S8 and S9):* Increased pressure in the proximal aorta was induced by means of thoracic aortic banding as described [59]. Male mice (C57BL/6, 6-8 weeks old) were anesthetized with ketamine (100 mg/kg) and xylazine (5 mg/kg), intubated and attached to a ventilator (Harvard Apparatus) at 100 breaths per minute. An incision was made in the chest wall approximately at the third intercostal space. The transverse aortic arch was ligated (7-0 silk) between the innominate and left common carotid arteries with an overlaying of a blunted 27-gauge needle, and then the needle was removed, leaving a discrete region of stenosis. The chest was closed, and the lung re-inflated. Mice were given analgesics and monitored for recovery.

*Heart weight and histological analysis after aorta-banding (Figs S8 and S9):* The banded mice were euthanized 3 weeks after operation. Total body weight was measured, and the heart was removed. The heart was blotted dry and weighed. The heart was then cut in half, fixed in 10% neutral buffered formalin, and processed for paraffin sections. Tissue sections were stained with 0.1% Sirius red in picric acid. Perivascular fibrosis index (a percent ratio of area of perivascular fibrosis to vascular luminal area) and myocardial total collagen content (a percent rate of Sirius-red stained collagen area to total myocardial area in microscopic field) were assessed in digitized microscopic images.
