# Supplementary figures and images for: Extracellular BCL2 Proteins Are Danger-Associated Molecular Patterns That Reduce Tissue Damage in Murine Models of Ischemia-Reperfusion Injury
Source: PLoS One. 2010 Feb 8;5(2):e9103. doi: 10.1371/journal.pone.0009103 (PMC2816997; doi:10.1371/journal.pone.0009103)

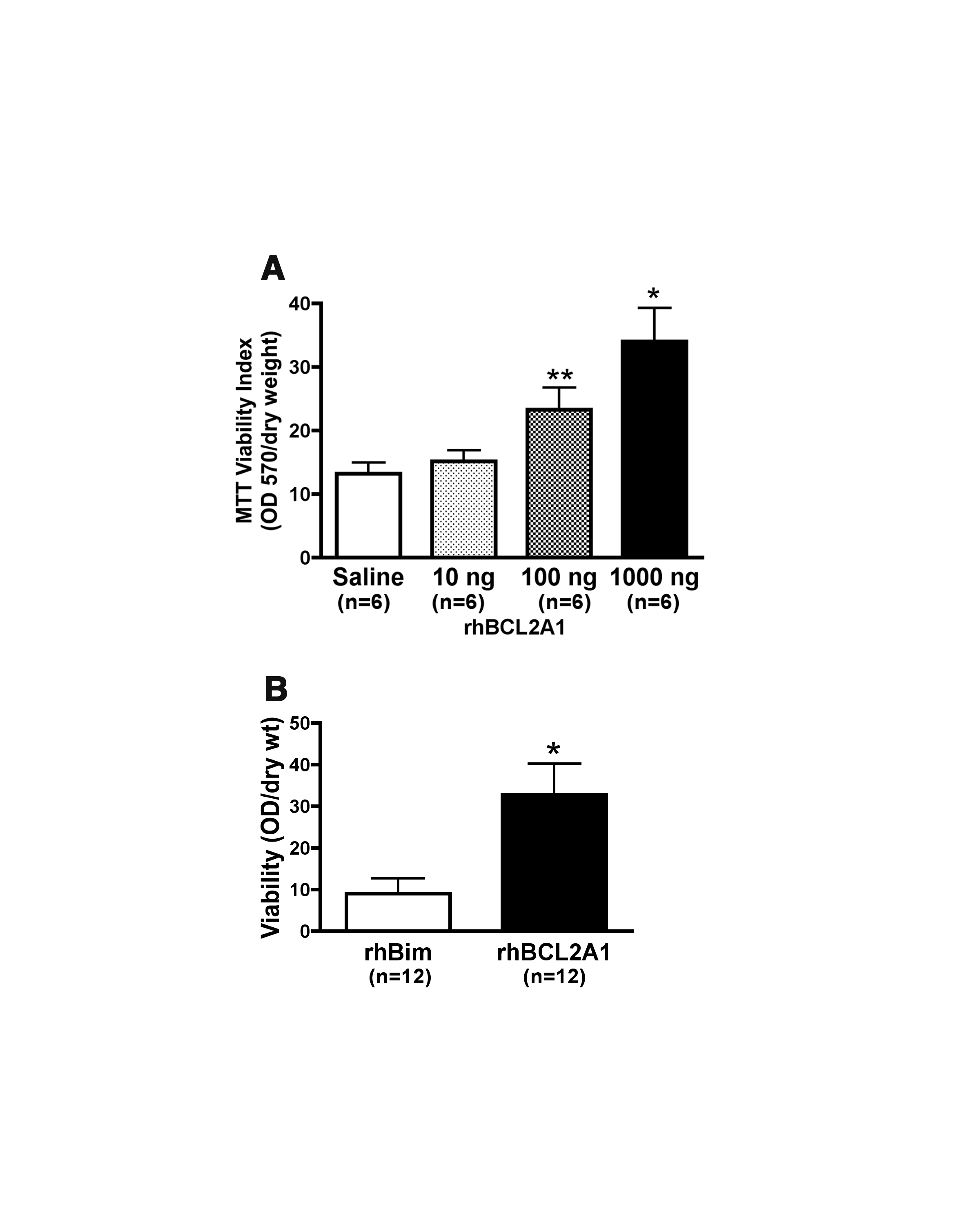

Supplement: Figure S1 — Protection against I/R injury by rhBCL2A1 is dose-dependent and persists for up to 72 hours. (A) Mice were subjected to tourniquet ischemia for 90 minutes, and treated with subcutaneous rhBCL2A1 (1 Î¼g/mouse) at the time of reperfusion. Tissue viability, measured the following day by MTT assay, was increased by treatment with rhBCL2A1 in dose-dependent manner (*p<0.01 vs. saline, **p<0.05 vs. saline). (B) Mice were treated with subcutaneous rhBCL2A1 or rhBim (1 Î¼g/mouse) at 72 hrs prior to 90 minutes of ischemia followed by 24 hours of reperfusion. Tissue viability as measured by MTT assay was significantly greater in the rhBCL2A1-treated mice compared with the rhBim-treated mice (*p<0.05). (7.23 MB TIF) [file pone.0009103.s002.tif]

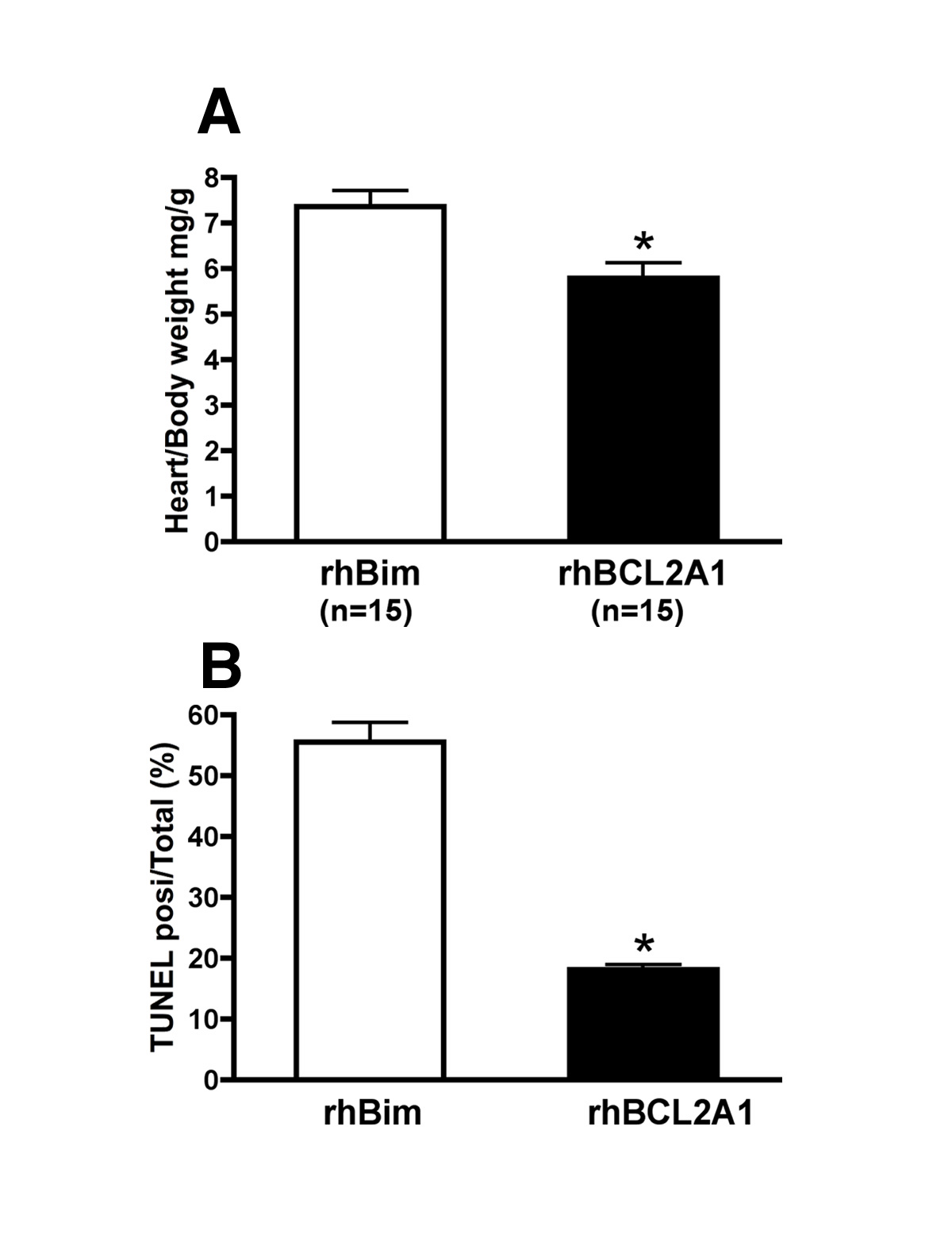

Supplement: Figure S8 — rhBCL2A1 reduces cardiac hypertrophy and apoptosis induced by aorta-banding. (A) Bar graph shows the effect of rhBCL2A1 treatment on heart weight/body weight ratio at 3 weeks after aorta-banding procedure. At the time of aorta-banding mice were injected subcutaneously with rhBCLA1 or rhBim (1Î¼g/mouse). (*p<0.05) (B) Bar graph showing the effect of rhBCL2A1 on DNA strand breaks by TUNEL staining of heart at 3 weeks after aorta-banding procedure. At the time of aorta-banding mice were injected subcutaneously with rhBCLA1 or rhBim (1Î¼g/mouse). (*p<0.05) (5.66 MB TIF) [file pone.0009103.s009.tif]

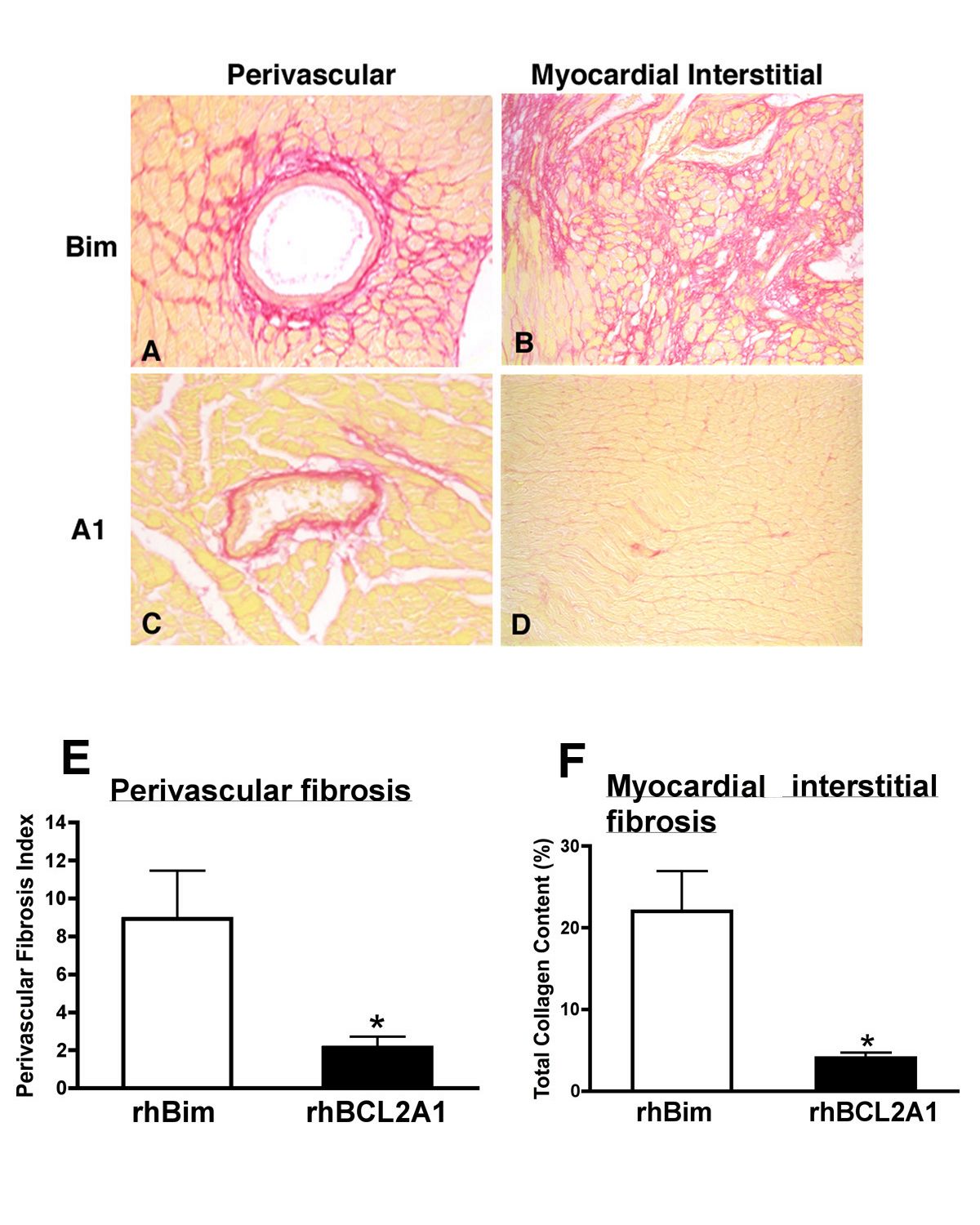

Supplement: Figure S9 — hBCL2A1 reduces development of cardiac fibrosis following aorta-banding. Histological sections of hearts were stained with Sirius-red to highlight collagen. (A, B) show hearts from rhBim-treated animal, (C, D) from rhBCL2A1-treated animal. (A, C) show perivascular fibrosis, and (B, D) show myocardial fibrosis. (original magnification: ×40). (E, F) show quantification by morphometry of the Sirius-red staining area. (E) illustrates perivascular fibrosis index as measured by perivascular collagen normalized to the vascular luminal area. (F) illustrates myocardial interstitial fibrosis determined as percent of collagen area to myocardial area in microscopic field. (*p<0.05) (5.43 MB TIF) [file pone.0009103.s010.tif]
